# Booster dose of COVID-19 vaccine and its effectiveness against post COVID-19 condition in individuals with pre-existing immunity: a sequential propensity score-matched cohort study

**DOI:** 10.1016/j.eclinm.2026.104215

**Published:** 2026-09-23

**Authors:** Lisa Morris, Erik Bülow, Huiqi Li, Yiyi Xu, Magnus Gisslén, Fredrik Nyberg, Maria Bygdell

**Affiliations:** a Department of Internal Medicine and Clinical Nutrition, Institute of Medicine, Sahlgrenska Academy, University of Gothenburg, Gothenburg, Sweden; b Unit of Clinical Pharmacology, Department of Pharmaceuticals, Sahlgrenska University Hospital, Region Västra Götaland, Gothenburg, Sweden; c School of Public Health and Community Medicine, Institute of Medicine, Sahlgrenska Academy, University of Gothenburg, Gothenburg, Sweden; d Department of Infectious Diseases, Institute of Biomedicine, Sahlgrenska Academy, University of Gothenburg, Gothenburg, Sweden; e Department of Infectious Diseases, Sahlgrenska University Hospital, Region Västra Götaland, Gothenburg, Sweden

**Keywords:** COVID-19, Post-COVID-19 condition, Long-COVID, COVID-19 vaccination, Propensity score matching

## Abstract

**Background:**

COVID-19 vaccination before primary infection is associated with decreased risk for post COVID-19 condition, but its effectiveness in populations with pre-existing immunity against SARS-CoV-2 is unknown. Our aim was to assess the COVID-19 vaccine effectiveness against post COVID-19 condition of four doses compared with three doses, both in the general population and in subpopulations with and without risk factors for severe acute COVID-19.

**Methods:**

In this, sequential, propensity score-matched cohort study conducted between 27 December 2020 and 29 February 2024, we included adults (≥18 years) who had received their third dose of COVID-19 vaccine, had no previous diagnosis of post COVID-19 condition, and who were successfully matched. At each index date (the date of the fourth dose for the exposed), an individual who had not yet received a fourth dose was included as unexposed, using exact matching followed by propensity score matching. Propensity scores were estimated based on socioeconomic factors, comorbidities, health-seeking behaviours, previous COVID-19, and prescribed medications. Individuals were followed from index date until diagnosis of post COVID-19 condition, moving out of study counties, death, new vaccination, six months after index date, or end of follow-up. The primary outcome was a clinical diagnosis of post COVID-19 condition from primary or specialist care.

**Findings:**

In total, 801,869 exposed individuals who received a fourth dose were successfully matched with 801,869 individuals with three doses. The incidence rate of post COVID-19 condition in the group with three doses was 1.67 per 1000 person-years (95% CI 1.52–1.83), compared with 1.14 per 1000 person-years (95% CI 1.02–1.28) in the four-dose group. In Cox regression analyses, a fourth vaccine dose was associated with a decreased risk of being diagnosed with post COVID-19 condition, with a vaccine effectiveness of 31% (95% CI 22–38). Among those with risk factors for severe acute COVID-19, the incidence rates of post COVID-19 condition were 2.14 per 1000 person-years (95% CI 1.91–2.39) for three doses and 1.32 per 1000 person-years (95% CI 1.07–1.54) for four doses, with a vaccine effectiveness of 38% (95% CI 29–47) for the fourth dose against post COVID-19 condition.

**Interpretation:**

Receiving a fourth dose of COVID-19 vaccine was associated with a lower risk of being diagnosed with post COVID-19 condition compared with three doses, although the absolute risk reduction was small. Due to the observational design of the study, residual confounding cannot be excluded.

**Funding:**

Swedish state under the agreement between the Swedish government and the county councils (the ALF-agreement), the Swedish Research Council, King Gustaf V's and Queen Victoria's Foundation, the SciLifeLab from the Knut and Alice Wallenberg Foundation, the Swedish Research Council for Health, Working Life, and Welfare (FORTE), the Swedish Society for Medical Research (SSMF), and the Swedish Research Council for Environment, Agricultural Sciences and Spatial Planning (FORMAS).

**Research in context**

**Evidence before this study**

We searched PubMed on 1 June 2026, using the terms (“post COVID-19 condition” OR “long COVID”) AND (“vaccine” OR “vaccination”) AND (“pre-existing immunity” OR “booster”) without language restriction. We found 73 articles that were screened based on title and abstract. After this screening, we identified one systematic review evaluating the impact of a booster dose on post COVID-19 condition. A third or fourth dose was associated with reduced odds of developing post COVID-19 condition compared to primary vaccination series only (defined as one or two doses). However, the certainty of evidence of the pooled result was considered very low. Additonally, we found four other studies in different settings using surveys that had evaluated the vaccine effectiveness against post COVID-19 condition in individuals with pre-existing immunity. Large, population-based studies are rare and it is not known if the potential protection against post COVID-19 condition differs between individuals with and without risk factors for severe acute COVID-19.

**Added value of this study**

Our findings of a protective effect against post COVID-19 condition add to the evidence of the effectiveness of the COVID-19 vaccines regarding prolonged effects from the infection. We show that receiving a fourth vaccine dose was associated with a decreased risk of being diagnosed with post COVID-19 condition, compared to three doses, during the first 6 months after booster vaccination, in a population with pre-existing immunity against SARS-CoV-2. The effectiveness was higher among individuals with risk factors for severe acute COVID-19 compared to those without.

**Implications of all the available evidence**

Our findings show that there is still some additional vaccine protection against post COVID-19 condition of receiving a fourth dose compared to three doses in a population with pre-existing immunity, but the absolute risk reduction is limited. The protection was higher in the risk group for severe acute COVID-19 and lower in the non-risk group. These positive effects from the COVID-19 vaccines could be taken into account when planning vaccination strategies for individuals. More research on the potential beneficial effects of the vaccines against prolonged symptoms should be conducted in other contexts and using different measures of post COVID-19 condition.

## Introduction

Since its emergence, COVID-19 has had profound global health consequences, causing acute suffering, illness, and death across populations. As the pandemic unfolded, it quickly became evident that the infection could lead to long-term health complications, including newly developed post-acute conditions or persistent symptoms such as fatigue, dyspnoea, anosmia, cardiac abnormalities, and cognitive impairment. The estimated prevalence of persistent symptoms after COVID-19 differs depending on study populations and designs, but WHO estimates that 6% of individuals with COVID-19 are affected by long-term symptoms.^1^ Although the WHO no longer considers acute COVID-19 a public health emergency of international concern, the long-term effects of the disease can still be a burden on individuals and continue to strain both healthcare systems and societies. As SARS-CoV-2, the virus causing COVID-19, continues to circulate and cause recurrent infections, the risk of post COVID-19 condition following acute COVID-19 persists, although its incidence appears to have declined over time with increasing population immunity and a lower proportion of severe acute cases.

From a public health perspective, it is important to evaluate preventive measures for post COVID-19 condition to mitigate its individual and societal burden. If administered before primary infection, COVID-19 vaccination has been shown to reduce the risk of developing post COVID-19 condition in an apparent dose–response manner (one to three doses before first infection).^2^^,^^3^ However, most individuals have now developed some degree of immunity against SARS-CoV-2, through previous infections and vaccinations. In this context, it remains uncertain whether COVID-19 vaccination confers additional protection against post COVID-19 condition in a population with pre-existing immunity to the virus. A recent systematic review including a meta-analysis indicated that receiving a third or fourth dose, compared to primary vaccination series only (defined as one or two doses), was associated with reduced odds of developing post COVID-19 condition, with a pooled odds ratio of 0.77. However, important factors such as post COVID-19 condition definitions, follow-up periods, and time since last vaccination differed between the three included studies, and the certainty of evidence of the pooled result was considered very low.^4^ Furthermore, it is not known if the potential protection against post COVID-19 condition differs between individuals with and without risk factors for severe acute COVID-19.

In Sweden, the International Classification of Diseases 10th revision (ICD-10) diagnosis code for post COVID-19 condition was implemented clinically in October 2020,^5^ creating an opportunity to study key epidemiological aspects of post COVID-19 condition using registers. The National Board of Health and Welfare recommends the diagnosis code to be used by physicians when a condition is assessed to be associated with a previous COVID-19. Using the unique Swedish personal identification number (PIN), information on individuals can be linked across several national health, disease, and sociodemographic registers with high linkage quality.^6^ In the present population-based register study, we had access to data covering approximately 40% of the Swedish population aiming to investigate COVID-19 vaccine effectiveness against post COVID-19 condition in a population with pre-existing immunity to SARS-CoV-2, more reflective of the current immunity landscape. Specifically, our aim was to assess the effectiveness against post COVID-19 condition of a fourth vaccine dose compared with three doses, both in the general population and in subpopulations with and without risk factors for severe acute COVID-19.

## Methods

### Study design and data sources

This sequential propensity score-matched cohort study used data from the Swedish COVID-19 Investigation for Future Insights—a Population Epidemiology Approach using Register Linkage (SCIFI-PEARL) project. SCIFI-PEARL is a register-based population cohort in Sweden with comprehensive data collected from a unique linkage of several national, regional, and quality registers using the Swedish PIN.^7^ The data originate from the National Register of Notifiable Diseases (SmiNet),^8^ the National Vaccination Register, the National Patient Register (NPR),^9^ the regional primary healthcare databases in Stockholm county (VAL) and Västra Götaland county (VEGA), the Swedish Intensive Care Register (SIR), the National Prescribed Drug Register, the Multi-Generation Register, the National Register of Care and Social Services for the Elderly and Persons with Impairments (SOL), and the Longitudinal Integrated Database for Health Insurance and Labour Market Studies (LISA).^10^ SmiNet includes all positive SARS-CoV-2 polymerase chain reaction (PCR) test results, the National Vaccination Register comprises all administered COVID-19 vaccinations, NPR covers all inpatient and specialist physician outpatient healthcare visits, the VAL and VEGA databases include both public and most private primary healthcare contacts in Stockholm and Västra Götaland regions (the two largest regions in Sweden with nearly 40% of the Swedish population^11^), SIR comprises all intensive care stays, the National Prescribed Drug Register contains all prescribed drugs dispensed at pharmacies, the Multi-Generation Register includes information on connections between biological and adoptive parents and children, SOL includes social care interventions provided under the Swedish Social Services Act, and the LISA database covers demographic and socioeconomic data.

### Ethics

The study was conducted in accordance with the Declaration of Helsinki and was approved by the Swedish Ethical Review Authority (decision no. 2020-01800 with subsequent amendments). Informed consent was waived as the study was based on pseudonymized register data.

### Source population and study population

The source population included all adults (≥18 years of age) residing in Stockholm or Västra Götaland county at the latest registration before administration of the first vaccine dose in Sweden (27 December 2020), and who received a third dose of COVID-19 vaccine between 27 December 2020 and 29 February 2024 (end of study). The study population included individuals from the source population who were successfully matched (see below) and had no diagnosis of post COVID-19 condition before their index (matching) date.

### Covariates for propensity score estimation

Information on year of birth, age group (18–64 or ≥65 years of age), gender (men or women), residential county (Stockholm/Västra Götaland), urbanicity, country of birth (Sweden/not Sweden), parents’ country of birth, education, occupation, income, and marital status (married/not married) were all obtained from the LISA database. Urbanicity was defined according to specific geographical areas (demographic statistical areas, DeSO) and categorised as rural, suburban, or urban areas. Parents’ country of birth was defined as both parents from Sweden, one parent from Sweden, or no parent from Sweden. Education was divided into four categories: pre-secondary (<10 years), secondary (10–12 years), higher (>12 years), or unknown. Occupation was categorised as healthcare worker, other essential worker (including teachers, service sector workers, police and security services, postal and delivery workers, cleaners, and taxi, bus, and tram drivers), non-essential worker, or unemployed. Income levels were grouped into four quartiles based on the distribution of disposable income in Sweden. Information on adoption was obtained from the Multi-Generation Register, and information on nursing home residence and home care services was gathered from SOL. The number of healthcare contacts in 2019 was used as a surrogate for health-seeking behaviour and was categorised as 0, 1–3, 4–10, and >10 contacts. A healthcare contact was defined as any registered contact with primary healthcare, specialist outpatient care, or specialist inpatient care. If more than one contact occurred on the same day, this was counted as a single contact. Likewise, each inpatient period was defined as one contact only. Pregnancy was defined as having an ICD-10 diagnosis code for delivery (O80–O84) recorded in the NPR, VEGA, or VAL within nine months after the third dose. COVID-19 before the third vaccine dose was defined by a positive PCR-test result in SmiNet, or a clinical ICD-10 diagnosis code (U07.1 or U07.2) in VEGA, VAL, NPR, or SIR and classified as no registered infection, non-hospitalised infection, or hospitalised infection. Hospitalisation was defined as having a primary diagnosis of COVID-19 in the inpatient part of NPR or in SIR. Comorbidities and medication use were defined using relevant ICD-10 diagnosis codes and Anatomical Therapeutic Chemical (ATC) codes (Supplementary Table S1). Antihypertensive and antidepressant treatment were also included as covariates to complement diagnostic data, while immunosuppressants, antineoplastic agents, and systemic corticosteroids were used to define immunosuppression treatment. Having at least one risk factor for severe acute COVID-19 (yes or no) was defined as being 65 years of age or older, living in a nursing home or special facility for the elderly, having home care services, being pregnant, or having comorbidities including diabetes, cardiovascular disease, chronic respiratory disease, obesity diagnosis, renal failure, liver disease, Down's syndrome, neuromuscular disease, or immunodeficiency due to medication or underlying disease. These were all considered to be risk factors for severe acute COVID-19 by the Public Health Agency in Sweden during the study period. Age group, gender, and having at least one risk factor for severe acute COVID-19 were, together with the week of the third dose, used as strata within which the propensity score matching was performed. All other variables described were included in the propensity score model.

### Matching procedure and exposure

Propensity scores for the exposure (a fourth vaccine dose) were estimated for each individual, including a large set of variables as described above (Table 1). Individuals who received a fourth dose of a COVID-19 vaccine were considered exposed, and the date of the fourth dose was set as the index date. These individuals were propensity score matched 1:1, greedy, sequential, and without replacement to unexposed individuals who had not received a fourth dose up until the index date of their matched exposed counterpart. The unexposed individuals were assigned the same index date as their exposed match. Thus, a later exposed individual could potentially earlier also serve as an unexposed control and thereby contribute unexposed person-time up to receiving their fourth dose. The propensity score matching was performed within strata based on gender (men and women), age group (18–64 and ≥ 65 years of age), week of the third vaccine dose, and having at least one risk factor for severe acute COVID-19 (yes and no). For each exposed individual, candidate controls from the corresponding risk set were evaluated based on the squared difference in the Cox model linear predictor for exposure. Matched pairs were retained only if this distance was below 0.2 times the standard deviation of the pairwise distance distribution.

**Table 1:** Characteristics of the matched study population, by exposure status.

Characteristics	3 doses (unexposed) n = 801,869	4 doses (exposed) n = 801,869	SMD
Having risk factors for severe acute COVID-19^a^	486,195 (61%)	486,195 (61%)	0
Year of birth, median (IQR)	1959 (1948–1970)	1959 (1947–1971)	0
Age group			0
18–64 years	446,330 (56%)	446,330 (56%)	0
≥65 years	355,539 (44%)	355,539 (44%)	0
Gender			0
Men	363,055 (45%)	363,055 (45%)	0
Women	438,814 (55%)	438,814 (55%)	0
Residential county			0.06
Stockholm	451,563 (56%)	474,969 (59%)	0.06
Västra Götaland	350,306 (44%)	326,900 (41%)	0.06
Urbanicity			0.02
Rural	87,760 (11%)	82,949 (10%)	0.02
Suburban	35,535 (4.4%)	34,681 (4.3%)	0.01
Urban	678,574 (85%)	684,239 (85%)	0.02
Adopted	13,698 (1.7%)	14,977 (1.9%)	0.01
Swedish-born	682,358 (85%)	684,977 (85%)	0.01
Parents' country of birth			0.02
Both from Sweden	708,967 (88%)	705,033 (88%)	0.02
One parent from Sweden	59,867 (7.5%)	62,797 (7.8%)	0.01
No parent from Sweden	33,035 (4.1%)	34,039 (4.2%)	0.01
Education^b^			0.02
Pre-secondary	113,758 (14%)	116,063 (14%)	0.01
Secondary	297,661 (37%)	294,170 (37%)	0.01
Higher	383,239 (48%)	383,436 (48%)	0
Unknown	7211 (0.9%)	8200 (1.0%)	0.01
Occupation			0.05
Unemployed	237,217 (30%)	245,966 (31%)	0.02
Non-essential worker	421,156 (53%)	424,133 (53%)	0.01
Other essential worker^c^	83,540 (10%)	81,399 (10%)	0.01
Healthcare worker	59,956 (7.5%)	50,371 (6.3%)	0.05
Income levels			0.02
Quartile 1	102,373 (13%)	108,073 (13%)	0.02
Quartile 2	164,719 (21%)	165,348 (21%)	0
Quartile 3	195,298 (24%)	190,959 (24%)	0.01
Quartile 4	339,479 (42%)	337,489 (42%)	0.01
Marital status			0.02
Married	418,728 (52%)	409,440 (51%)	0.02
Not married	383,141 (48%)	392,429 (49%)	0.02
Living in a nursing home	9970 (1.2%)	9135 (1.1%)	0.01
Living with home care services	29,745 (3.7%)	33,067 (4.1%)	0.02
Number of healthcare contacts in 2019			0.01
0	117,262 (15%)	115,236 (14%)	0.01
1–3	194,657 (24%)	192,752 (24%)	0.01
4–10	242,807 (30%)	241,396 (30%)	0
>10	247,143 (31%)	252,485 (31%)	0.01
Pregnant	3137 (0.4%)	3062 (0.4%)	0
Comorbidities^d^			
Cardiovascular disease	256,673 (32%)	253,467 (32%)	0.01
Chronic respiratory disease	66,190 (8.3%)	65,774 (8.2%)	0
Diabetes	60,407 (7.5%)	61,373 (7.7%)	0
Psychiatric disorders	161,082 (20%)	163,174 (20%)	0.01
Obesity diagnosis	40,068 (5.0%)	39,738 (5.0%)	0
Liver disease	5796 (0.7%)	5672 (0.7%)	0
Renal failure	18,226 (2.3%)	19,048 (2.4%)	0.01
Down's syndrome	558 (<0.1%)	786 (<0.1%)	0.01
Neuromuscular disease	1179 (0.1%)	1266 (0.2%)	0
Immunosuppression due to disease	3048 (0.4%)	3170 (0.4%)	0
Antihypertensive treatment	301,897 (38%)	304,182 (38%)	0.01
Antidepressant treatment	108,497 (14%)	113,154 (14%)	0.02
Immunosuppression due to treatment	89,489 (11%)	90,342 (11%)	0
COVID-19 related characteristics			
Registered COVID-19 before dose 3^e^			0.04
No	673,847 (84%)	663,367 (83%)	0.04
Yes, hospitalised	5614 (0.7%)	6653 (0.8%)	0.01
Yes, non-hospitalised	122,408 (15%)	131,849 (16%)	0.03

SMD = Standardised mean difference.

All variables listed were included in the propensity score matching procedure. Values are numbers (percentages) unless stated otherwise. The source population included all adult residents in Stockholm and Västra Götaland counties in Sweden who had received a third vaccine dose between 27 December 2020 and 29 February 2024.

a Defined as being ≥65 years of age, living in a nursing home or special facility for the elderly, having home care services, being pregnant, or having comorbidities including diabetes, cardiovascular disease, chronic respiratory disease, obesity diagnosis, liver disease, renal failure, Down's syndrome, neuromuscular disease, or immunodeficiency due to medication or underlying disease.

b Pre-secondary: <10 years, Secondary: 10–12 years, Higher: >12 years.

c Including teachers, service sector workers, police and security services, postal and delivery workers, cleaners, and taxi, bus, and tram drivers.

d Comorbidities registered in VEGA, VAL, or NPR between 2015 and 2019. Medications registered in National Prescribed Drug Register between 2018 and 2019.

e COVID-19 defined as either a positive PCR-test result in SmiNet or a clinical diagnosis code (U07.1 or U07.2) in VEGA, VAL, NPR, or SIR.

### Outcome and follow-up

The outcome was defined as a registered post COVID-19 condition diagnosis (ICD-10 code U09.9) from primary or specialist healthcare. Each individual in the matched pair was followed from their index date until the earliest of a post COVID-19 condition diagnosis, or censoring due to new vaccination, moving out of Stockholm or Västra Götaland county, death, 6 months after index date, or end of study (29 February 2024), contributing person-time until that date. The whole matched pair was censored if an individual was censored due to new vaccination, to avoid potential imbalance between the groups due to informative censoring. This censoring approach was evaluated in sensitivity analyses.

### Statistics

Descriptive statistics are presented as median and IQR for continuous variables and as counts and percentages for categorical variables. For the urbanicity variable, missing data were handled using multiple imputation by chained equations, while missing information for the education variable was coded as a separate category (unknown). Propensity scores for the exposure were estimated using a large set of covariates (Table 1) in a Cox proportional hazards regression model. Continuous variables were modelled using natural cubic splines, and selected interaction terms were incorporated to allow for effect modification. Standardised mean differences (SMD) were calculated for all variables used in the calculation of propensity scores and were used to assess sample balance after matching and to quantify potential differences among the three-dose and four-dose groups. An SMD less than 0.1 was considered acceptable balance between groups. Incidence rates and incidence rate differences were estimated, and 95% CIs were obtained using nonparametric bootstrap with 200 resamples at the matched-pair level. Kaplan Meier failure probability was used to illustrate the cumulative incidence of post COVID-19 condition among unexposed and exposed individuals. Cox proportional hazards regression models were used to estimate hazard ratios (HR) with 95% CIs, for four versus three doses of COVID-19 vaccine. The vaccine effectiveness against post COVID-19 condition for a fourth vaccine dose was calculated as (1-HR) ∗ 100. The proportional hazards assumption was ascertained through visual inspection of Schoenfeld residual plots and by the Grambsch–Therneau test based on the Schoenfeld residuals. Statistical significance was defined as a p-value of less than 0.05.

We used SAS version 9.4 for data management and R version 4.4.1 for statistical analyses.

To evaluate effect modification, we included an interaction term between the exposure variable and having or not having at least one risk factor for severe acute COVID-19. We then performed separate stratified analyses to evaluate the vaccine effectiveness against post COVID-19 condition in individuals with risk factors for severe acute COVID-19, and in those without.

We performed several sensitivity analyses evaluating the robustness of the study design. The first analysis was performed using matching with replacement. The second set of sensitivity analyses evaluated different approaches to avoid potential imbalance between groups caused by informative censoring; (1) application of inverse probability censoring weights and (2) no censoring for new vaccination during follow-up in any group. The third set of sensitivity analyses aimed to increase the sensitivity and specificity, respectively, of the outcome measure post COVID-19 condition; (1) including the ICD-10 diagnosis code for post-viral fatigue (G93.3) together with post COVID-19 condition, creating a broader composite outcome variable to increase the sensitivity and (2) excluding individuals who had been diagnosed with other severe acute respiratory infections in specialist care within 3 months before or within 6 months after their index date, to increase the specificity, since post-infectious conditions can arise from many different infections. The respiratory infections were defined by ICD-10 diagnosis codes retrieved from the NPR (Supplementary Table S1). If an individual was excluded due to a previous severe acute respiratory infection, their matched counterpart was also excluded from the analysis. Finally, in the last sensitivity analysis, individuals with an index date before 1 April 2022 were excluded to analyse the vaccine effectiveness against post COVID-19 condition likely related to infections occurring during the Omicron era.

### Role of funding source

The funders had no role in study design, data collection, data analyses, data interpretation, or the writing of the report.

## Results

During the study period, 801,869 adults (74% of the source population who received a fourth vaccine dose) were propensity score matched to 801,869 adults with three doses at equivalent time points, forming the final study population (Supplementary Figure S1). The study population included a total of 1,385,497 unique individuals, where some appeared in both groups, being selected as matched unexposed comparators (three doses) before they became exposed (four doses) and then also selected as exposed. Of the remaining 26% of the source population with four vaccine doses, 239,499 individuals (22%) were included in the three-dose group only before receiving their fourth vaccine dose, while in total 45,710 individuals (4%) were excluded due to; a prior diagnosis of post COVID-19 condition (n = 6082), no subsequent match (n = 4548), or not fulfilling the matching criteria (n = 35,080; Supplementary Figure S1). The three-dose and four-dose groups included in the study were completely balanced regarding their exact matching variables, and variables used in the propensity score matching were similar between groups (all SMD <0.1; Table 1). Similarly, the distribution of the propensity scores had completely overlapping range between the groups after matching (Supplementary Figure S2). The most commonly used vaccine brands for dose four are listed in Supplementary Table S2. The median follow-up was 120 days (IQR 28–180) for the three-dose group and 121 days (28–180) for the four-dose group.

The incidence rate of post COVID-19 condition among those with three doses was 1.67 per 1000 person-years (95% CI 1.52–1.83), compared with 1.14 per 1000 person-years (95% CI 1.02–1.28) among those with four doses, resulting in an incidence rate difference of −0.52 (95% CI −0.74 to −0.33) per 1000 person-years (Table 2). The results are also illustrated in a plot of the Kaplan–Meier failure probability (Fig. 1). In a Cox proportional hazards regression, receiving a fourth dose of a COVID-19 vaccine was associated with a decreased risk of receiving a post COVID-19 condition diagnosis as compared with three doses (HR 0.69, 95% CI 0.62–0.78) with a vaccine effectiveness of 31% (95% CI 22–38; Table 3).

**Table 2:** Estimated risk of post COVID-19 condition among individuals with three (unexposed) or four (exposed) vaccine doses in the total study population and stratified by having or not having risk factors for severe acute COVID-19.

	3 doses, PCC (n)/Total person-years	4 doses, PCC (n)/Total person-years	3 doses, incidence rate (95% CI)^a^^,^^b^	4 doses, incidence rate (95% CI)^a^^,^^b^	Incidence rate difference (95% CI)^a^^,^^b^
Total population	388/234,283	268/234,852	1.67 (1.52, 1.83)	1.14 (1.02, 1.28)	−0.52 (−0.74, −0.33)
Risk group^c^	241/111,637	147/112,213	2.14 (1.91, 2.39)	1.32 (1.07, 1.54)	−0.82 (−1.21, −0.50)
No risk group	147/122,646	121/122,639	1.20 (1.02, 1.38)	0.98 (0.81, 1.17)	−0.21 (−0.45, 0.04)

PCC = post COVID-19 condition; CI = confidence interval.

Counts, incidence rates, and incidence rate differences of PCC displayed. The source population included all adult residents in Stockholm and Västra Götaland counties in Sweden who had received a third vaccine dose between 27 December 2020 and 29 February 2024.

a Per 1000 person-years.

b Confidence intervals estimated using nonparametric bootstrap with 200 resamples at the matched-pair level.

c Defined as being ≥65 years of age, living in a nursing home or special facility for the elderly, having home care services, being pregnant, or having comorbidities including diabetes, cardiovascular disease, chronic respiratory disease, obesity diagnosis, liver disease, renal failure, Down's syndrome, neuromuscular disease, or immunodeficiency due to medication or underlying disease.

**Fig. 1:**
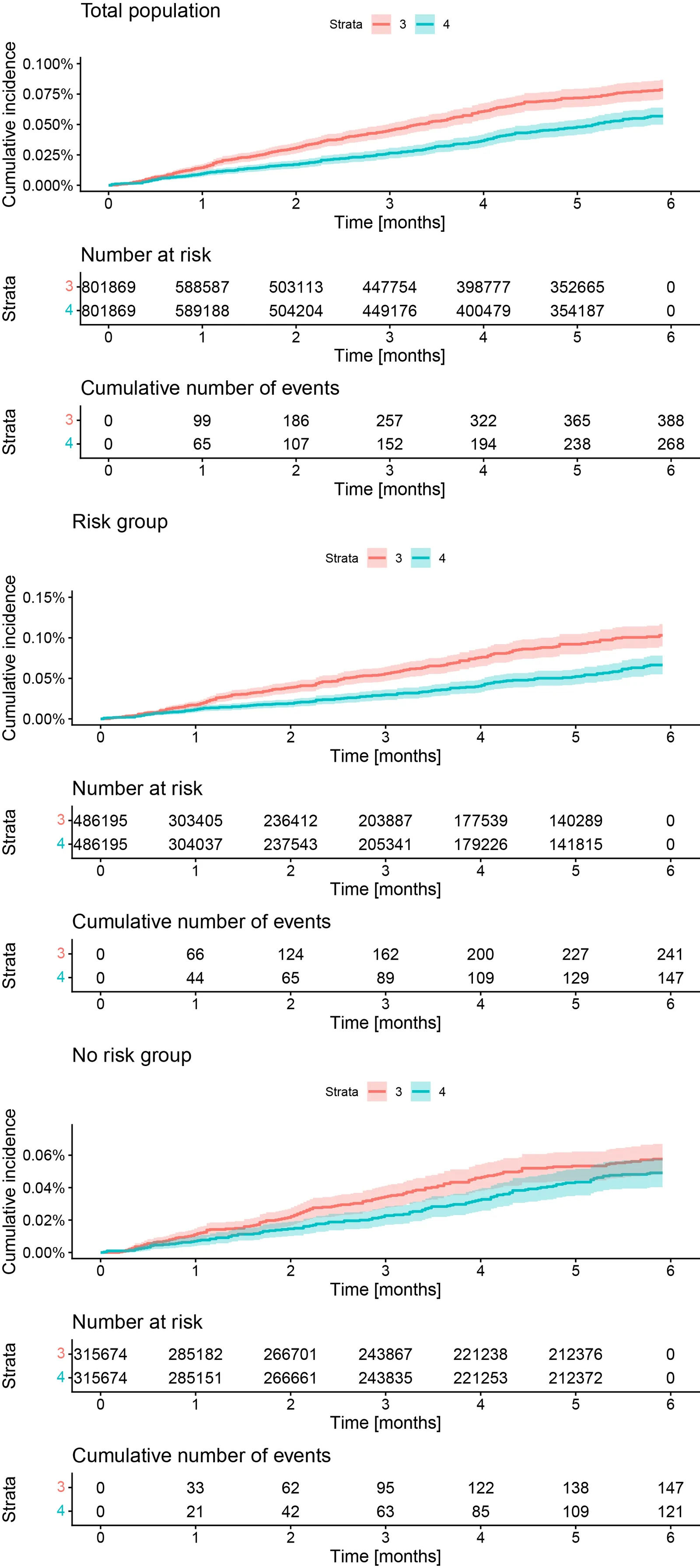
Kaplan Meier failure probability of post COVID-19 condition in the total study population and stratified by having or not having risk factors for severe acute COVID-19. (Risk group defined as being ≥65 years of age, living in a nursing home or special facility for the elderly, having home care services, being pregnant, or having comorbidities including diabetes, cardiovascular disease, chronic respiratory disease, obesity diagnosis, liver disease, renal failure, Down's syndrome, neuromuscular disease, or immunodeficiency due to medication or underlying disease.) The source population included all adult residents in Stockholm and Västra Götaland counties in Sweden who had received a third vaccine dose between 27 December 2020 and 29 February 2024.

**Table 3:** The association between receiving a fourth vaccine dose and being diagnosed with post COVID-19 condition in the total study population and stratified by having or not having risk factors for severe acute COVID-19.

	Hazard ratio (95% CI)	Vaccine effectiveness (95% CI)
Total population	0.69 (0.62–0.78)	31% (22–38)
Risk group^a^	0.62 (0.53–0.71)	38% (29–47)
No risk group	0.82 (0.69–0.98)	18% (2–31)

CI = confidence interval.

Hazard ratios and vaccine effectiveness (95% confidence intervals) displayed. The source population included all adult residents in Stockholm and Västra Götaland counties in Sweden who had received a third vaccine dose between 27 December 2020 and 29 February 2024.

a Defined as being ≥65 years of age, living in a nursing home or special facility for the elderly, having home care services, being pregnant, or having comorbidities including diabetes, cardiovascular disease, chronic respiratory disease, obesity diagnosis, liver disease, renal failure, Down's syndrome, neuromuscular disease, or immunodeficiency due to medication or underlying disease.

Inclusion of an interaction term between vaccination status and having or not having ≥1 risk factor for severe acute COVID-19 into the model resulted in a statistically significant (p-value <0.05) interaction indicating effect modification. In separate stratified analyses, the incidence rate difference between the three-dose and four-dose groups was statistically significant among those with risk factors for severe acute COVID-19 (−0.82, 95% CI −1.21 to −0.50), but not among those without (−0.21, 95% CI −0.45 to 0.04; Table 2). Among those with risk factors, the vaccine effectiveness of a fourth dose was 38% (95% CI 29–47), while the corresponding effectiveness was 18% (95% CI 2–31) among those without (Table 3).

In a sensitivity analysis using replacement in the matching process, 1,072,873 individuals (99% of the source population who received a fourth dose) could be matched and were included, forming a new study population (Supplementary Figure S3). After matching, the resulting three- and four-dose groups were similar to each other (Supplementary Table S3). The incidence rate difference of post COVID-19 condition between the groups, and the vaccine effectiveness, were essentially the same as in the main analysis without replacement (Supplementary Tables S4 and S5).

In sensitivity analyses evaluating different approaches to compensate for potential imbalance between groups caused by informative censoring, the results were mainly consistent with the results from the main analysis, although the estimated vaccine effectiveness was slightly lower in some analyses compared to the main analysis. For example, when applying inverse probability censoring weights, the vaccine effectiveness did not reach statistical significance among individuals without risk factors for severe acute COVID-19 and when no censoring for vaccination was applied, the vaccine effectiveness against post COVID-19 condition was lower in the general population and in subgroups compared to the results in the main analysis (Supplementary Tables S4 and S5).

In the sensitivity analysis using a composite outcome (post COVID-19 condition and post-viral fatigue) to increase the sensitivity of the outcome, the estimated vaccine effectiveness was somewhat lower than in the main analysis, while the incidence rate differences between the three-dose and four-dose groups were slightly larger and statistically significant in the general population as well as in both subgroups (Supplementary Tables S4 and S5).

In the sensitivity analysis excluding individuals who had been diagnosed with other severe acute respiratory infections, to increase the specificity of the outcome, the results were similar to the results in the main analysis (Supplementary Tables S4 and S5).

In the sensitivity analysis including only those with an index date from 1 April 2022 and onwards (851 matched pairs excluded), the findings were similar to the main analysis (Supplementary Tables S4 and S5).

## Discussion

In a population with pre-existing immunity against SARS-CoV-2, we show that receiving a fourth vaccine dose was associated with a decreased risk of being diagnosed with post COVID-19 condition, compared to three doses, during the first 6 months after booster vaccination, corresponding to a vaccine effectiveness of 31%. However, the absolute risk reduction was very small, with an incidence rate difference of only −0.52 per 1000 person-years between the four- and three-dose groups. The effectiveness was higher among individuals with risk factors for severe acute COVID-19 compared to those without, with a vaccine effectiveness of 38% versus 18%, respectively. The incidence rate difference between the three- and four-dose groups was −0.82 per 1000 person-years in the risk group and −0.21 per 1000 person-years in the non-risk group.

Although the pandemic phase has passed, SARS-CoV-2 continues to circulate in society, resulting in repeated exposures and highlighting the need to clarify the implications of reinfections. A register-based study from the US Veterans Affair database showed that reinfections were associated with an increased risk of death, hospitalisation, and organ-specific sequelae compared to no reinfection, in a population with a high prevalence of established risk factors for severe disease.^12^ These results suggest that reinfections still have an impact on health outcomes, in both the acute and post-acute phase. As long as SARS-CoV-2 circulates in society, individuals are at risk of developing acute infection and potentially post COVID-19 condition. Therefore, evaluating vaccine effectiveness and appropriate targeting of preventive measures against COVID-19 and post COVID-19 condition remains important. SARS-CoV-2 has continued to evolve, giving rise to multiple variants, with Omicron subvariants now dominating. These variants seem to differ in their ability to cause post COVID-19 condition, and studies indicate that post COVID-19 condition is less common after Omicron infection than after earlier variants.^13^^,^^14^ Several explanations have been proposed, including differences in virus characteristics that may influence its potential to cause post COVID-19 condition, as well as pre-existing immunity in the population resulting from prior SARS-CoV-2 infections and vaccinations. The roll-out of the fourth vaccine dose in Sweden started in February 2022, with a fourth dose being available to everyone over 18 years from September 2022.^15^ With Omicron being the most predominant virus variant from January 2022 and onwards, the absolute majority of the post COVID-19 condition diagnoses in our study are likely to stem from Omicron infections.

COVID-19 vaccination before primary SARS-CoV-2 infection has been shown to reduce the risk of post COVID-19 condition.^2^^,^^3^ However, whether additional booster doses in populations with pre-existing immunity against SARS-CoV-2 protects against post COVID-19 condition, is essentially unknown. A recent systematic review and meta-analysis including three studies that compared a booster dose (third or fourth dose) with a primary vaccination series (defined as one or two doses) before Omicron infection showed a reduction in the odds of developing post COVID-19 condition.^4^ However, the certainty of evidence was considered very low due to the study designs and inconsistency between the studies regarding post COVID-19 condition definitions, follow-up periods, types of vaccines used, and time since vaccination. Furthermore, all three studies relied on self-reported data regarding both vaccination status and post COVID-19 condition symptoms.^16^, ^17^, ^18^ The Public Health Agency of Sweden performed a health economic analysis in 2025 on the benefits of continued COVID-19 vaccination in the population.^19^ They concluded that the societal costs for vaccination are reasonable for older individuals, especially for those who are at risk for severe acute disease due to having risk factors, while it appear less reasonable for individuals without risk factors for severe acute disease, or for younger individuals in risk groups. However, they state that the effect from post COVID-19 condition is not included in their analyses, and that the vaccine effectiveness against post COVID-19 condition today is uncertain. Most studies evaluating the vaccine effectiveness against post COVID-19 condition do not report the effect separately for individuals with and without risk factors for severe acute COVID-19. Hence, evidence remains scarce on the vaccine effectiveness against post COVID-19 condition in these specific groups. A Swedish cohort study recently showed that receiving two or three vaccine doses was associated with a reduced risk of being diagnosed with persistent post COVID-19 condition compared with being unvaccinated, in individuals both below and above 65 years of age (which is considered the age cut-off for increased risk of severe acute disease).^20^ In the present study, we analysed the vaccine effectiveness against post COVID-19 condition in two separate groups; individuals with risk factors for severe acute COVID-19 (including several relevant risk factors in addition to age) and individuals without. Our results show a statistically significant protective effect of a fourth dose against post COVID-19 condition in individuals with risk factors and a more modest protective effect in those without. The low number of post COVID-19 condition diagnoses during follow-up among individuals without risk factors for severe acute disease may have contributed to the protective effect in this group not reaching statistical significance in all sensitivity analyses.

While COVID-19 vaccines remain highly effective at preventing severe acute disease, the protection against infection has weakened over time, although they still offer some protection.^21^, ^22^, ^23^ It is plausible that the vaccine-induced protection against post COVID-19 condition can, at least in part, be attributed to the protection against SARS-CoV-2 infection itself as well as the protection against severe acute disease, given that infection is a prerequisite for post COVID-19 condition and severe acute disease is a well-established risk factor for long-term symptoms.^24^ In our earlier work, we demonstrated that vaccination before primary infection is associated with a reduced risk of post COVID-19 condition not only among individuals with severe acute disease but also among non-hospitalised individuals,^2^ suggesting that additional mechanisms or pathways may contribute to the protective effect. In the present study, infection was not required for inclusion, and the observed protection against post COVID-19 condition is therefore likely to reflect the vaccines’ capacity to reduce the risk of acute infection and severe disease, while not excluding the contribution of additional mechanisms. Furthermore, part of the observed difference between the groups might also stem from waning of the protective effect against post COVID-19 condition conferred by the third dose, contributing to more post COVID-19 condition cases within the three-dose group.

The present study has many strengths: use of data from high-quality Swedish registers linked by the unique Swedish PIN with high accuracy, the large sample size, the population-based data, and the robustness of the analyses demonstrated with several relevant sensitivity analyses. Furthermore, we have access to primary healthcare data, which is crucial when performing register-based research on post COVID-19 condition in a Swedish setting since post COVID-19 condition is a condition mainly managed in primary healthcare. Since it is mandatory and regulated by law to register all administered vaccine doses in the National Vaccination Register, the risk for misclassification of the exposure is considered negligible. The socioeconomic and demographic variables used in the propensity score matching were obtained from high-quality national registers with low risk of misclassification, but the comorbidity variables used were obtained from healthcare registers meaning that some misclassification might occur (as seeking healthcare and receiving a diagnosis are required for the condition to be registered). However, we do not believe that this potential misclassification would differ systematically between the three-dose and four-dose groups. A limitation of the present study is that the diagnosis code for post COVID-19 condition is not yet formally validated, which may result in misclassification of the outcome. However, we believe that this is largely unrelated to the exposure and should therefore affect both groups similarly and would thus tend to bias results toward the null. This is important to note, since the true effect of vaccination might be somewhat stronger than our results. Another limitation is that we do not have information on specific post COVID-19 condition symptoms and cannot differentiate between cases with mild, isolated symptoms and those with more severe manifestations of post COVID-19 condition. We also only capture individuals who have sought healthcare for their symptoms and received a diagnosis of post COVID-19 condition, probably missing some milder cases. Physicians might also have problems distinguishing post COVID-19 condition from other post-viral conditions during the study period, since full-population testing recommendations for COVID-19 were terminated in Sweden 9 February 2022. However, our sensitivity analysis evaluating a composite outcome including post COVID-19 condition and the general diagnosis for post-viral fatigue provided essentially the same results as the main analysis including only post COVID-19 condition as the outcome, indicating that this is not a critical concern. Lastly, since this is an observational study, residual confounding and healthy vaccine bias cannot fully be ruled out, although we do not include any unvaccinated individuals in our study and most of the individuals in the three-dose group eventually receive a fourth dose.

In conclusion, we demonstrate that receiving a fourth dose of COVID-19 vaccine is associated with a decreased risk of being diagnosed with post COVID-19 condition during the first 6 months after vaccination, although the incidence rate difference was small. The protective effect against post COVID-19 condition was observed in individuals both with and without risk factors for severe acute COVID-19, with higher effect in the at-risk group and lower effect in the group without risk factors.

## Contributors

The work described in this article was conceptualised and designed by LM, MG, FN, and MB. All authors made substantial contributions to the literature search (LM, MB) acquisition of data (HL, YX, FN), data curation (HL, EB), data analysis (EB), preparation of figures (EB), or interpretation of data (LM, EB, HL, YX, MG, FN, and MB). LM and MB drafted the manuscript, all authors revised it critically for important intellectual content, and LM and MB subsequently edited the manuscript. All authors read and gave their approval of the final version to be published and agree to be accountable for all aspects of the work, ensuring that questions related to the accuracy or integrity of any part of the work are appropriately investigated and resolved. More than one author has directly accessed and verified the underlying data reported in the manuscript (EB, FN, MB).

## Data sharing statement

The data used in this study are pseudonymized and sourced from Swedish healthcare registers and are not publicly available according to Swedish legislation. Interested researchers may seek ethical approval to access data from the appropriate Swedish public data holders according to the relevant legislation.

## Declaration of interests

HL reports participation in research (regulator-mandated phase IV study; investigator-initiated study) with support from Bayer, AstraZeneca (to their institution, no personal fees), with no relation to the work reported here, and is a member of executive committee in the Nordic PharmacoEpidemiological Network (NorPEN). MG has received research grants from Gilead Sciences and honoraria as speaker, DSMB committee member and/or scientific advisor from Amgen, AstraZeneca, Biogen, Bristol-Myers Squibb, CSI Seqirus, Gilead Sciences, GlaxoSmithKline/ViiV, Janssen-Cilag, MSD, Novocure, Novo Nordic, Pfizer and Sanofi (outside of submitted work). FN reports participation in research (regulator-mandated phase IV study; investigator-initiated study) with support from Bayer, AstraZeneca (to their institution, no personal fees), with no relation to the work reported here. FN previously owned some AstraZeneca shares. LM has received travel grants for conference attendance from the Sahlgrenska Academy, the Gothenburg Medical Society, and the Herbert and Karin Jacobson Foundation. The other authors declare no competing interests.
