# Supplementary material for: Booster dose of COVID-19 vaccine and its effectiveness against post COVID-19 condition in individuals with pre-existing immunity: a sequential propensity score-matched cohort study
Source: eClinicalMedicine. 2026 Sep 23;100:104215. doi: 10.1016/j.eclinm.2026.104215 (PMC13634098; doi:10.1016/j.eclinm.2026.104215)
Supplement: Supplementary file 1 [file mmc1.docx]

**Supplemental material**

**Booster dose of COVID-19 vaccine and its effectiveness against post COVID-19 condition in individuals with pre-existing immunity: a sequential propensity-scored matched cohort study**

**Table S1.** ICD-10 and ATC codes used to define comorbidities and medication use.

| **Comorbidities^*^** | **ICD-10 codes** |
| --- | --- |
| Cardiovascular disease | I00-I99 |
| Chronic respiratory disease | J40-J99 |
| Diabetes | E10, E11 |
| Psychiatric disorders | F00-F99 |
| Obesity diagnosis | E66 |
| Liver disease | K70-K77 |
| Renal failure | N17-N19 |
| Down’s syndrome | Q90 |
| Neuromuscular disease | G12, G70, G71 |
| Immunosuppression due to disease | D80-D89 |
| Severe acute respiratory infection^†^ | J03-J06, J09-J18, J20-J22, J85-J86 |
| **Prescribed medications^‡^** | **ATC codes** |
| Antihypertensive treatment | C02, C03A, C03EA01, C07 (not C07AA07), C08C, C09A-D, C09X |
| Antidepressant treatment | N06A |
| Immunosuppression due to treatment | L04, L01, H02A |

ICD-10 = International Classification of Diseases, 10th Revision; ATC = Anatomical Therapeutic Chemical classification.

^*^Comorbidities registered as primary or secondary diagnoses in VEGA, VAL or NPR between 2015-2019. NPR includes both specialist inpatient and outpatient healthcare, VEGA and VAL include primary healthcare data from physicians and non-physicians.

^†^ICD-10 diagnosis codes registered in the NPR 3 months prior to index date or within 6 months after index date.

**^‡^** Medications registered in the National Prescribed Drug Register between 2018-2019.

**Table S2.** The five most common vaccines used for the fourth dose.

| **Fourth vaccine dose** | **n (%)** |
| --- | --- |
| BNT162b2^*^ | 620,706 (77) |
| mRNA-1273^†^ | 180,812 (23) |
| NVX-CoV2373**^‡^** | 346 (0.04) |
| AZD1222**^§^** | 4 (<0.01) |
| Ad26.COV2-S^‖^ | 1 (<0.01) |

^*^ Comirnaty, Pfizer/BioNTech

^†^ Spikevax, Moderna

**^‡^** Nuvaxovid, Novavax CZ

**^§^** Vaxzevria, Oxford-Astra Zeneca

^‖^ Jcovden, Janssen-Cilag

**Table S3.** Characteristics of the matched study population when propensity score matching was performed with replacement, by exposure status. Values are numbers (percentages) unless stated otherwise. The source population included all adult residents in Stockholm and Västra Götaland counties in Sweden who had received a third vaccine dose between 27 December 2020 and 29 February 2024.

| **Characteristics** | **3 doses (unexposed)**  **n = 1,072,878** | **4 doses (exposed)** **n = 1,072,878** | **SMD** |
| --- | --- | --- | --- |
| Having risk factors for severe acute COVID-19^*^ | 739,347 (69) | 739,347 (69) | 0 |
| Year of birth, median (q1, q3) | 1955 (1945, 1966) | 1955 (1945, 1967) | 0 |
| Age group |  |  | 0 |
| 18-64 | 483,265 (45) | 483,265 (45) | 0 |
| ≥65 | 589,613 (55) | 589,613 (55) | 0 |
| Gender |  |  | 0 |
| Men | 486,220 (45) | 486,220 (45) | 0 |
| Women | 586,658 (55) | 586,658 (55) | 0 |
| Residential county |  |  | 0.02 |
| Stockholm | 596,626 (56) | 608,179 (57) | 0.02 |
| Västra Götaland | 476,252 (44) | 464,699 (43) | 0.02 |
| Urbanicity |  |  | 0.01 |
| Rural | 117,980 (11) | 118,953 (11) | 0 |
| Suburban | 47,304 (4.4) | 48,939 (4.6) | 0.01 |
| Urban | 907,594 (85) | 904,986 (84) | 0.01 |
| Adopted | 17,240 (1.6) | 19,562 (1.8) | 0.02 |
| Swedish-born | 913,496 (85) | 921,185 (86) | 0.02 |
| Parents’ country of birth |  |  | 0.02 |
| Both from Sweden | 962,010 (90) | 954,000 (89) | 0.02 |
| One parent from Sweden | 72,301 (6.7) | 77,911 (7.3) | 0.02 |
| No parent from Sweden | 38,567 (3.6) | 40,967 (3.8) | 0.01 |
| Education^†^ |  |  | 0.01 |
| Pre-secondary | 172,341 (16) | 171,410 (16) | 0 |
| Secondary | 403,249 (38) | 400,821 (37) | 0 |
| Higher | 487,489 (45) | 490,501 (46) | 0.01 |
| Unknown | 9,799 (0.9) | 10,146 (0.9) | 0 |
| Occupation |  |  | 0.03 |
| Unemployed | 380,973 (36) | 381,987 (36) | 0 |
| Non-essential worker | 517,467 (48) | 522,267 (49) | 0.01 |
| Other essential worker^‡^ | 99,424 (9.3) | 101,595 (9.5) | 0.01 |
| Healthcare worker | 75,014 (7.0) | 67,029 (6.2) | 0.03 |
| Income levels |  |  | 0 |
| Quartile 1 | 150,250 (14) | 150,812 (14) | 0 |
| Quartile 2 | 239,275 (22) | 238,582 (22) | 0 |
| Quartile 3 | 248,032 (23) | 248,843 (23) | 0 |
| Quartile 4 | 435,321 (41) | 434,641 (41) | 0 |
| Marital status |  |  | 0.01 |
| Married | 567,828 (53) | 562,102 (52) | 0.01 |
| Not married | 505,050 (47) | 510,776 (48) | 0.01 |
| Living in a nursing home | 15,834 (1.5) | 15,637 (1.5) | 0 |
| Living with home care services | 50,258 (4.7) | 51,565 (4.8) | 0.01 |
| Number of healthcare contacts in 2019 |  |  | 0.02 |
| 0 | 137,428 (13) | 133,066 (12) | 0.01 |
| 1-3 | 246,314 (23) | 241,841 (23) | 0.01 |
| 4-10 | 333,467 (31) | 332,017 (31) | 0 |
| >10 | 355,669 (33) | 365,954 (34) | 0.02 |
| Pregnant | 3,306 (0.3) | 3,237 (0.3) | 0 |
| **Comorbidities^§^** | | | |
| Cardiovascular disease | 390,020 (36) | 389,195 (36) | 0 |
| Chronic respiratory disease | 94,836 (8.8) | 96,801 (9.0) | 0.01 |
| Diabetes | 90,985 (8.5) | 92,635 (8.6) | 0.01 |
| Psychiatric disorders | 215,392 (20) | 223,689 (21) | 0.02 |
| Obesity diagnosis | 55,104 (5.1) | 56,475 (5.3) | 0.01 |
| Liver disease | 8,517 (0.8) | 8,416 (0.8) | 0 |
| Renal failure | 28,549 (2.7) | 29,578 (2.8) | 0.01 |
| Down’s syndrome | 728 (< 0.1) | 909 (< 0.1) | 0.01 |
| Neuromuscular disease | 1,616 (0.2) | 1,765 (0.2) | 0 |
| Immunosuppression due to disease | 4,074 (0.4) | 4,400 (0.4) | 0 |
| Antihypertensive treatment | 458,239 (43) | 460,452 (43) | 0 |
| Antidepressant treatment | 145,096 (14) | 154,634 (14) | 0.03 |
| Immunosuppression due to treatment | 126,165 (12) | 130,343 (12) | 0.01 |
| **COVID-19 related characteristics** | | | |
| Registered COVID-19 before dose 3^‖^ |  |  | 0.04 |
| No | 926,192 (86) | 911,842 (85) | 0.04 |
| Yes, hospitalised | 7,889 (0.7) | 9,022 (0.8) | 0.01 |
| Yes, non-hospitalised | 138,797 (13) | 152,014 (14) | 0.04 |

SMD=Standardised mean difference.

**^*^**Defined as being ≥65 years of age, living in a nursing home or special facility for the elderly, having home care services, being pregnant, or having comorbidities including diabetes, cardiovascular disease, chronic respiratory disease, obesity diagnosis, liver disease, renal failure, Down’s syndrome, neuromuscular disease, or immunodeficiency due to medication or underlying disease.

^†^Pre-secondary: <10 years, Secondary: 10-12 years, Higher: >12 years.

^‡^Including teachers, service sector workers, police and security services, postal and delivery workers, cleaners, and taxi, bus, and tram drivers.

**^§^**Comorbidities registered in VEGA, VAL or NPR between 2015-2019. Medications registered in the National Prescribed Drug Register between 2018-2019.

^‖^COVID-19 defined as either a positive PCR-test result in SmiNet or a clinical diagnosis code (U07.1 or U07.2) in VEGA, VAL, NPR, or SIR.

**Table S4.** Estimated risk of post COVID-19 condition among individuals with three (unexposed) or four (exposed) vaccine doses in the total study population and stratified by having or not having risk factors for severe acute COVID-19. Counts, incidence rates, and incidence rate differences of PCC displayed. Analyses performed with propensity score matching with replacement, followed by propensity score matching without replacement with two different censoring approaches (censoring based on inverse probability censoring weights and no censoring at new vaccination), followed by analyses of a composite outcome including PCC and post-viral fatigue, followed by exclusion of individuals with severe acute respiratory infection, and by exclusion of individuals with an index date prior to 1 April 2022. The source population included all adult residents in Stockholm and Västra Götaland counties in Sweden who had received a third vaccine dose between 27 December 2020 and 29 February 2024.

|  | **3 doses**  **PCC (n) /**  **Total person-years** | **4 doses**  **PCC (n) /**  **Total person- years** | **3 doses Incidence rate (95%CI)^*^**^†^ | **4 doses Incidence rate (95%CI)^*^**^†^ | **Incidence rate difference (95%CI)^*^**^†^ |
| --- | --- | --- | --- | --- | --- |
| **Matching with replacement** | | | | | |
| **Total population** | 495/  306,887 | 312/  308,039 | 1.61  (1.49, 1.73) | 1.01  (0.91, 1.11) | -0.60  (-0.77, -0.43) |
| **Risk group^‡^** | 363/  178,383 | 198/  179,553 | 2.03  (1.84, 2.24) | 1.09  (0.95, 1.23) | -0.94  (-1.21, -0.67) |
| **No risk group** | 132/  128,504 | 114/  128,486 | 1.03  (0.84, 1.18) | 0.89  (0.72, 1.05) | -0.14  (-0.39, 0.11) |
| **Censoring using IPCW^§^**^¶^ | | | | | |
| **Total population** | 654/  365,415 | 279/  256,627 | 1.78  (1.55, 2.02) | 1.08  (0.96, 1.21) | -0.70  (-0.95, -0.44) |
| **Risk group^‡^** | 482/  222,516 | 157/  121,891 | 2.16  (1.83, 2.65) | 1.28  (1.09, 1.51) | -0.88  (-1.33, -0.46) |
| **No risk group** | 172/  142,898 | 123/  134,736 | 1.21  (1.01, 1.41) | 0.90  (0.72, 1.06) | -0.31  (-0.57, -0.07) |
| **No censoring at new vaccination^§^** | | | | | |
| **Total population** | 519/  383,544 | 413/  383,911 | 1.36  (1.24, 1.48) | 1.07  (0.97, 1.19) | -0.28  (-0.44, -0.13) |
| **Risk group^‡^** | 349/  234,836 | 270/  235,218 | 1.49  (1.35, 1.63) | 1.15  (1.02, 1.28) | -0.34  (-0.54, -0.17) |
| **No risk group** | 170/  148,708 | 143/  148,694 | 1.13  (0.99, 1.31) | 0.96  (0.82, 1.12) | -0.17  (-0.42, 0.03) |
| **Composite outcome (PCC and post-viral fatigue)^§^** | | | | | |
| **Total population** | 616/  232,485 | 456/  233,059 | 2.65  (2.44, 2.85) | 1.97  (1.81, 2.13) | -0.68  (-0.90, -0.43) |
| **Risk**  **group^‡^** | 356/  110,709 | 243/  111,285 | 3.21  (2.80, 3.54) | 2.19  (1.92, 2.45) | -1.02  (-1.44, -0.52) |
| **No risk group** | 260/  121,776 | 213/  121,774 | 2.12  (1.88, 2.37) | 1.74  (1.51, 1.97) | -0.38  (-0.67, -0.05) |
| **Excluding individuals with severe acute respiratory infection^§^**^‖^ | | | | | |
| **Total population** | 364/  231,520 | 260/  232,030 | 1.57  (1.42, 1.72) | 1.12  (0.97, 1.24) | -0.46  (-0.65, -0.27) |
| **Risk**  **group^‡^** | 220/  109,514 | 139/  110,032 | 2.02  (1.75, 2.25) | 1.27  (1.07, 1.46) | -0.75  (-1.04, -0.39) |
| **No risk group** | 144/  122,006 | 121/  121,998 | 1.18  (1.00, 1.42) | 0.99  (0.84, 1.16) | -0.19  (-0.44, 0.08) |
| **Excluding individuals with an index date prior to 1 April 2022^§^** | | | | | |
| **Total population** | 387/  234,050 | 268/  234,619 | 1.65  (1.48, 1.81) | 1.14  (1.01, 1.29) | -0.50  (-0.72, -0.30) |
| **Risk**  **group**^‡^ | 240/  111,425 | 147/  112,000 | 2.14  (1.90, 2.44) | 1.30  (1.05, 1.51) | -0.83  (-1.21, -0.46) |
| **No risk group** | 147/  122,626 | 121/  122,618 | 1.19  (1.03, 1.37) | 0.97  (0.80, 1.12) | -0.23  (-0.50, 0.01) |

PCC=post COVID-19 condition; CI=confidence interval; IPCW=inverse probability censoring weights.

^*^per 1,000 person-years

^†^Confidence intervals estimated using nonparametric bootstrap with 200 resamples at the matched-pair level.

**^‡^** Defined as being ≥65 years of age, living in a nursing home or special facility for the elderly, having home care services, being pregnant, or having comorbidities including diabetes, cardiovascular disease, chronic respiratory disease, obesity diagnosis, liver disease, renal failure, Down’s syndrome, neuromuscular disease, or immunodeficiency due to medication or underlying disease.

**^§^**Propensity score matching performed without replacement.

^¶^Counts and person-years are multiplied by 1/censoring weights for each period and individual.

^‖^ICD-10 diagnosis codes (J03-J06, J09-J18, J20-J22, J85-J86) registered in the NPR 3 months prior to index or within 6 months after index date.

**Table S5.** The association between receiving a fourth vaccine dose and being diagnosed with post COVID-19 condition in the total study population and stratified by having or not having risk factors for severe acute COVID-19. Hazard ratios and vaccine effectiveness (95% confidence intervals) displayed. Analyses performed with propensity score matching with replacement, followed by propensity score matching without replacement with two different censoring approaches (censoring based on inverse probability censoring weights and no censoring at vaccination), followed by a composite outcome including PCC and post-viral fatigue, followed by exclusion individuals with severe acute respiratory infection, and by exclusion of individuals with an index date prior to 1 April 2022. The source population included all adult residents in Stockholm and Västra Götaland counties in Sweden who had received a third vaccine dose between 27 December 2020 and 29 February 2024.

|  | **Hazard Ratio (95% CI)** | **Vaccine effectiveness**  **% (95% CI)** |
| --- | --- | --- |
| **Matching with replacement** | | |
| **Total population** | 0.68 (0.60, 0.76) | 32 (24, 40) |
| **Risk group^*^** | 0.60 (0.51, 0.70) | 40 (30, 49) |
| **No risk group** | 0.83 (0.70, 0.99) | 17 (1, 30) |
| **Censoring using IPCW^†^** | | |
| **Total population** | 0.63 (0.57, 0.71) | 37 (29, 43) |
| **Risk group**^*^ | 0.55 (0.48, 0.63) | 45 (37, 52) |
| **No risk group** | 0.86 (0.72, 1.04) | NA |
| **No censoring at new vaccination^†^** | | |
| **Total population** | 0.80 (0.73, 0.87) | 20 (13, 27) |
| **Risk group^*^** | 0.78 (0.70, 0.86) | 22 (14, 30) |
| **No risk group** | 0.84 (0.72, 0.98) | 16 (2, 28) |
| **Composite outcome (PCC and post-viral fatigue)^†^** | | |
| **Total population** | 0.74 (0.68, 0.81) | 26 (19, 32) |
| **Risk group^*^** | 0.69 (0.61, 0.77) | 31 (23, 39) |
| **No risk group** | 0.82 (0.72, 0.93) | 18 (7, 28) |
| **Exclusion of individuals with severe acute respiratory infection^†‡^** | | |
| **Total population** | 0.72 (0.64, 0.81) | 28 (19, 36) |
| **Risk group ^*^** | 0.64 (0.55, 0.74) | 36 (26, 45) |
| **No risk group** | 0.84 (0.71, 0.998) | 16 (0, 29) |
| **Exclusion of individuals with an index date prior to 1 April 2022^†^** | | |
| **Total population** | 0.70 (0.62, 0.78) | 30 (22, 38) |
| **Risk group^*^** | 0.62 (0.53, 0.72) | 38 (28, 47) |
| **No risk group** | 0.82 (0.69, 0.98) | 18 (2, 31) |

CI=confidence interval; IPCW=inverse probability censoring weights; NA=not applicable; PCC=post COVID-19 condition.

^*^Defined as being ≥65 years of age, living in a nursing home or special facility for the elderly, having home care services, being pregnant, or having comorbidities including diabetes, cardiovascular disease, chronic respiratory disease, obesity diagnosis, liver disease, renal failure, Down’s syndrome, neuromuscular disease, or immunodeficiency due to medication or underlying disease.

**^†^** Propensity score matching performed without replacement

**^‡^**ICD-10 diagnosis codes (J03-J06, J09-J18, J20-J22, J85-J86) registered in the NPR 3 months prior to index or within 6 months after index date

**Supplemental figures**

**Figure S1.** Flow chart of the matching procedure showing individuals matched as unexposed (three doses) and/or exposed (four doses). The blue square represents the number of individuals included in the four-dose group and the sum of the red squares represents the number of individuals included in the three-dose group. The source population included all adult residents in Stockholm and Västra Götaland counties in Sweden who had received a third vaccine dose between 27 December 2020 and 29 February 2024.

**Figure S2**. Frequency plots of the propensity scores before (upper) and after matching (lower). The red curve represents the three-dose group (unexposed), and the blue curve represents the four-dose group (exposed). In the graph of the source population, individuals who changed exposure status from unexposed to exposed during the study period are represented in the exposed group only. Note that the propensity scores are calculated using Cox proportional hazards regression analyses, which affects the scale of the x-axis.

**Figure S3.** Flow chart of the matching procedure when using matching with replacement, showing individuals matched as unexposed (three doses) and/or exposed (four doses). The blue square represents the number of individuals included in the four-dose group and the sum of the red squares represents the number of individuals included in the three-dose group. The source population included all adult residents in Stockholm and Västra Götaland counties in Sweden who had received a third vaccine dose between 27 December 2020 and 29 February 2024.
